# Protective role of *Bergenia ciliata* in artesunate-induced nephrotoxicity: a translational pharmacology study integrating *in-silico* and experimental evidence

**DOI:** 10.3389/fphar.2026.1862707

**Published:** 2026-06-24

**Authors:** Sudip Prasad Jena, Sabina Evan Prince

**Affiliations:** School of Biosciences and Technology, VIT University, Vellore, Tamil Nadu, India

**Keywords:** Artesunate, *Bergenia ciliata*, GC-MS, molecular docking, network pharmacology, *in-vivo* study

## Abstract

**Introduction:**

Artesunate (ART) is a widely used antimalarial agent; however, dose-dependent nephrotoxicity limits its clinical utility. Bergenia Ciliata, a medicinal plant with established renoprotective traditional use, was investigated as a potential nephroprotective agent against ART-induced kidney injury.

**Methods:**

Bioactive metabolites from B. ciliata root extract were identified via Gas Chromatography-Mass Spectrometry (GC-MS) and filtered through in silico ADME profiling. Network pharmacology was employed to identify key hub genes implicated in ART-induced renal injury, with PPARγ emerging as the primary therapeutic target. Molecular docking and 200 ns molecular dynamics simulations were performed to evaluate binding affinity and complex stability. Cytotoxicity was assessed using the MTT assay on HEK293 cells. In vivo nephroprotective activity was evaluated in Wistar albino rats administered high-dose ART (150 mg/kg), with or without B. Ciliata root extract (200 and 400 mg/kg).

**Results:**

Rutin (CID 5280805) demonstrated the highest docking score (–10.307 kcal/mol) and binding affinity (MM-GBSA: –71.66 kcal/mol) against PPARγ, with stable complex dynamics confirmed over 200 ns. B. ciliata extract showed negligible cytotoxicity (>90% cell viability across all concentrations). In vivo, co-administration of B. Ciliata with ART significantly reduced elevated serum urea, uric acid, and creatinine levels in a dose-dependent manner, with the 400 mg/kg dose producing the most pronounced renoprotection. Histopathological evaluation confirmed measurable recovery of renal tissue architecture.

**Discussion:**

The integrated in-silico and experimental findings suggest that B. Ciliata root extract exerts nephroprotective effects against ART-induced kidney injury, likely through PPARγ activation mediated by rutin. The combination of B. Ciliata with ART demonstrated superior renoprotection compared to silymarin, supporting its potential as a complementary therapeutic strategy in high-dose antimalarial regimens.

## Graphical Abstract

**Figure:**
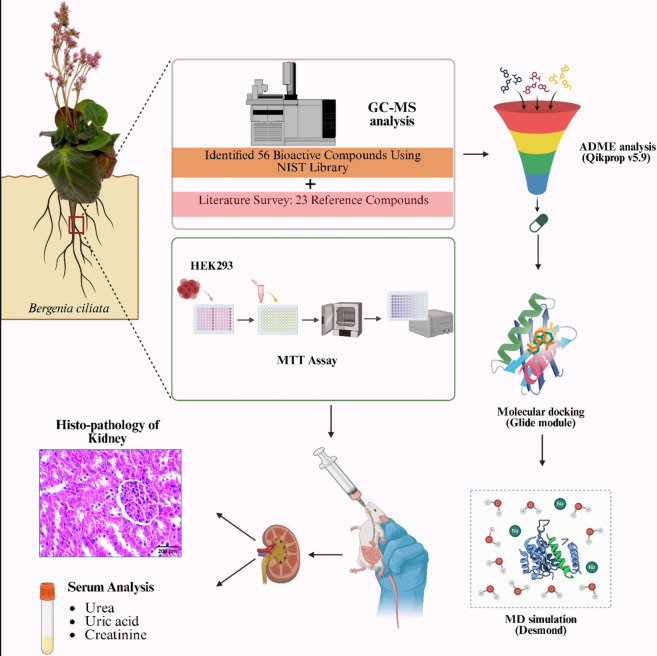
Created in BioRender. Ray, S. (2025) (https://BioRender.com/1124r3f).

## 1 Introduction

Synthetic and chemical drugs are presently facing numerous difficulties and issues, including the problem of developing chemo-resistance and producing side effects (George, 2011). Conversely, there has been a growing interest in the therapeutic potential of medicinal plants because they contain diverse phytochemicals with low toxicity (Nwozo et al., 2023). Many natural mixtures combined together as crude plant extracts are made up of a diverse assortment of the main bioactive components (Dar et al., 2023). Plant-derived chemicals such as saponins, alkaloids, terpenoids, flavonoids and tannins have demonstrated a broad array of biological activities, especially antifungal and antibacterial properties (Olivia et al., 2021). Identifying the primary bioactive chemical components of medicinal plants is essential, both to validate their traditional therapeutic applications and to provide structural leads for the development of clinically useful compounds. In the present study, Gas Chromatography-Mass Spectrometry (GC-MS) was employed to comprehensively profile the volatile and semi-volatile constituents present in the crude plant extract. GC-MS is a well-established hyphenated analytical technique that enables the separation, identification, and quantification of volatile phytochemical compounds by coupling the resolving power of gas chromatography with the structural elucidation capability of mass spectrometry, thereby allowing accurate characterization of the complex chemical composition of plant-derived extracts (Iordache et al., 2009).

Network pharmacology is an exciting discipline located at a confluence of pharmacology, systems biology, and networks (Z et al., 2012). Instead of studying one drug or target in isolation, it looks at the linkages between drug-in-trial, target-containing cells or tissues, and disease in order to see how all three relate on a systemic level (Agu et al., 2023). They give a strong foundation for clarifying molecular interactions, speeding up new medicines generation (Agu et al., 2023). By integrating drug-target interaction data, biological processes, and systems/network models researchers have gained new knowledge about mechanisms through which bioactive compounds act and how to identify the best targets for treatment (Singh et al., 2024).

The goal of the study was to determine how artesunate affects certain genes and to identify the possible phytochemical constituents in *Bergenia ciliata* root extract through both *in silico*, *in-vitro*, and *in-vivo* methods. The safety profile of *Bergenia ciliata* was assessed using an MTT assay at different concentrations. An *in silico* ADME (Absorption, Distribution, Metabolism, and Excretion) profile of selected compounds was performed to evaluate their safety and potential therapeutic efficacy, thereby identifying those with optimal drug-like properties and beneficial pharmacokinetic profiles. Network pharmacology was then used to evaluate and identify the top 10 hub genes of the network associated with therapeutic outcomes. PPARγ was found to be one of the top 10 hub genes related to kidney toxicity due to the inhibition of PPARγ by artesunate, which is an important protein involved in the repair mechanism of the kidney (Jang, 2016).

To evaluate the binding stability and interaction strength of the selected phytochemicals derived from Bergenia ciliata, the PPARγ receptor was subjected to molecular docking followed by molecular dynamics (MD) simulations. These computational analyses were performed to assess the conformational stability of the phytochemical-protein complex and to predict the potential of Bergenia ciliata bioactive constituents as PPARγ agonists capable of reversing ART-induced suppression of renal repair mechanisms. Subsequently, histopathological analysis of kidney tissue was conducted in rats subjected to high-dose artesunate (ART)-induced nephrotoxicity, with and without co-administration of Bergenia ciliata root extract (BCRE). This in vivo phase aimed to elucidate the renoprotective mechanisms underlying BCRE co-treatment, characterize the extent of structural recovery in renal tissue, and establish the therapeutic potential of Bergenia ciliata as a complementary agent in mitigating ART-induced kidney injury for future clinical applications.

## 2 Materials and methods

### 2.1 Identification of plant material and drug

Agri Exports is an authorized internet marketplace and has a source of *Bergenia ciliata* roots from Madhya Pradesh, India (Exports). Agri exports used the plant’s morphological characteristics to determine what the plant material was, and later identified by experts from the Xavier Research Foundation, St. Xavier’s College, Palayamkottai, Tamil Nadu. The plant specimen has been provided an accession number of XCH-40724. The online pharmacy purchased one Artesunate 200 mg Larinate®-200kit tablet.

### 2.2 Preparation and extraction of plant material

Fresh roots of *Bergenia ciliata* were washed under tap water for 10 min, followed by repeated rinsing with distilled water to remove impurities. The cleaned roots were dried under mild sunlight to preserve phytochemical integrity, chopped, and ground into fine powder using a mechanical grinder.

Extraction was carried out using a 1:20 (w/v) plant powder-to-solvent ratio with four polar solvents - ethanol, methanol, dichloromethane, and chloroform. The mixtures were agitated on a rotary shaker at room temperature for 48 h, then filtered through Whatman filter paper. The filtrates were evaporated at 40 °C–45 °C, and the resulting dry extracts were stored at 4 °C for further analysis.

### 2.3 Phytochemical analysis of *Bergenia ciliata* using gas chromatography-mass spectrometry (GC-MS)

Gas Chromatography Mass Spectrometry (GC/MS) was used to analyse the phytochemicals present in extracts of ethanol, Methanol, Dichloromethane and chloroform. Each extraction was filtered through a 0.2 μm polyvinylidene difluoride (PVDF) filter and diluted (10 mg) with its parent solvent (1 ml) prior to being injected into the GC/MS instrument after dilution and filtration. The injection port was maintained at 60 °C allowing for rapid volatilisation of the substances within the sample being analysed by helium as the carrier gas through the chromatographic column (Ghaoui et al., 1995). The stationary phase was made by utilizing materials that had been placed in the column. The way the compounds separated was based on how they interacted with the column and how much time they spent in the column. The oven that held the column was programmed to increase the temperature of the oven 10° per minute for 30 min and then to escalate the temperature from 30 to 260° over the next 30 min, which allowed compounds to elute from the column at progressively different times.

The compounds were identified as they exited the column using a mass spectrometer. Each peak on the chromatograms corresponded to a separate compound, with the height and area under the peak being directly related to the concentration of the respective compound in the sample. Compound identification was accomplished by comparing the mass spectrum obtained to the National Institute of Standards and Technology (NIST) library that is built into the GC/MS system software.

### 2.4 Network pharmacology and toxicology

#### 2.4.1 Target identification of artesunate

The potential molecular targets of Artesunate were identified using two established online databases: SwissTargetPrediction (Daina et al., 2019) and BATMAN-TCM (Liu et al., 2016). The SMILES (Simplified Molecular Input Line Entry System) representation of Artesunate, obtained from the PubChem database, was used as the input for both platforms.

#### 2.4.2 Identification of disease-associated genes

Genes associated with nephrotoxicity were retrieved from three publicly available databases: the Comparative Toxicogenomics Database (CTD) (Davis et al., 2026), GeneCards (Stelzer et al., 2016), and the Therapeutic Target Database (TTD) (Zhang et al., 2026). The search terms used included “Nephrotoxicity,” “Nephro Toxicity,” and “Kidney Injury.” The gene datasets obtained from the three databases were compiled into a single Excel file, and duplicate entries were removed to generate a unified list of disease-related genes. All data were retrieved in June 2025.

#### 2.4.3 Target overlap and protein–protein interaction (PPI) network construction

A Venn analysis was performed using Venny 2.1.0 (Oliveros, 2007) to identify common targets. Gene lists obtained from the three databases were input into the tool, and the intersecting genes common to all three datasets were identified. These overlapping genes were then imported into the STRING database (Szklarczyk et al., 2023) to construct a protein-protein interaction (PPI) network, enabling further exploration of the functional associations among the identified targets.

#### 2.4.4 Gene ontology

Gene Ontology (GO) analysis was performed using the DAVID database (Thomas et al., 2022) to identify potential therapeutic targets associated with biological processes (BP), cellular components (CC), and molecular functions (MF). Statistically significant GO terms were identified with a threshold of *P* < 0.05. Additionally, KEGG pathway analysis was conducted to evaluate the biological relevance of the identified therapeutic targets.

### 2.5 Protein and ligand preparation

The crystal structures of the top three hub genes implicated in kidney toxicity were retrieved from the Protein data bank with the following PDB IDs: 4R6E, 7U36, and 7AWC (Thorsell et al., 2017; Balboni et al., 2022; Willems et al., 2021). The retrieved structures contained unrelated elements, such as water molecules, heavy metals, ions, and co-crystallized ligands, that could interfere with the molecular docking process. These components were removed as part of the protein preparation protocol. Subsequently, hydrogen atoms were added, missing side chains were modelled and hydrogen bond orientations were optimized. The protein structures were then subjected to energy minimization using the OPLS force field. All pre-processing steps were carried out using the Protein Preparation Wizard in the Schrödinger Suite 2025.

For screening purposes, compounds were selected based on GC-MS analysis of *Bergenia ciliata* using the NIST library for compound identification, along with a literature survey of major phytochemicals previously reported in the plant, as mentioned in Table 2–6. A cumulative total of 79 chemical compounds were obtained in a 3-D structural representation (sdf format), the compounds were further pre-processed using the LigPrep module. Energy minimization was subsequently performed using the OPLS4 force field to optimize the geometry of the generated structures.

### 2.6 Binding site identification and grid generation

The top three hub genes, PARP1, GSK3β, and PPARγ, were identified based on the findings from the network pharmacology analysis. A subsequent literature review was conducted to determine each protein’s critical active site residues. For PARP1, the active site includes Gly863, Ser904, Tyr907, Tyr896, Lys903, Gly988, Leu769, Arg878, Ile879, Pro881, and Asp766 (Thorsell et al., 2017; Ferraris, 2010). The active site of GSK3β comprises Lys85, Asp200, Asp133, Val135, Glu137, Arg141, Gln185, and Arg220 (Balboni et al., 2022). In the case of PPARγ, the key residues forming the active site are Tyr473 and Arg288 (Willems et al., 2021). A grid box was created around the active site of all three hub genes using the grid generation tab in Schrodinger 2025, which enables ligand molecules to be screened out correctly.

### 2.7 Molecular docking and binding energy estimation

Following the successful preparation of proteins and ligands, along with grid generation, molecular docking studies were conducted to evaluate the binding potential of phytochemical compounds-identified through GC-MS analysis and a literature survey against the PPARγ proteins implicated in artesunate-induced nephrotoxicity. Docking was performed using the GLIDE module of Schrödinger Suite 2025.

We applied an efficient method of screening with an elaborate, three-step filtering process consisting of HTVS (High Throughput Virtual Screening), S.P. (Standard Precision), and XP (Extra Precision). In the initial step, ligand screening via HTVS and S.P. mode produced a very rapid ranking of the ligands based on their potential affinity via a common scoring method. After filtering the results produced in the first two steps, the ligands that were successful continued onto step three (XP docking). This step is a much more precise method of scoring because it uses a stricter scoring algorithm and an improved sampling procedure to produce an improved number of results with fewer false positives (Friesner et al., 2004).

### 2.8 Evaluation of the pharmacological similarities and ADME properties of the compounds assessed

We examined the ADME characteristics of the drugs with the help of the Qikprop v5.9 software distributed by Schrödinger 2025–1. The ADME characteristics were predicted using LigPrep Files and included a number of ranges of physicochemical parameters and significant descriptor values (Harder et al., 2016).

### 2.9 Molecular dynamics simulation

We employed the Desmond module of Schrödinger Suite 2025–1 to conduct a molecular dynamics (MD) simulation. The PPARγ-CID 5280805 complex was selected due to its high docking score and binding affinity (most successful of our screen). Prior to performing the simulation, the complex was prepared in an orthorhombic bounding box and solvated using a SPC (Single Point Charge) water model. A single Na^+^ was added to neutralize the complex, then NaCl was added to adjust ionic strength to simulate physiological salt levels of 0.15 M. The system was built using the OPLS4 force field and placed in an NPT ensemble at 300 K with 1.01 bar of pressure. The MD simulation lasted for 200 nanoseconds. After the work was done, the trajectory files were looked at to see how stable the structure of the protein-ligand complex was and how it moved.

### 2.10 Cell culture

Human Embryonic Kidney 293 (HEK293) cells were obtained from the National Centre for Cell Science (NCCS), Pune and grown in a humidified incubator at 37 °C, 5% CO_2_. The media used was 10% FBS and 1% antibiotic solution with 80%–90% confluence as the re-culture point.

### 2.11 MTT cell viability assay using HEK293 cells

This experiment employed the MTT assay to evaluate cell viability. Initially, cells were harvested and quantified using a hemocytometer. Subsequently, they were seeded into 96-well micro-titer plates at a density of 5,000 cells per well, with 100 µL of complete culture medium added to each well. The plates were incubated for 24 h at 37 °C in a 5% CO_2_ environment to facilitate proper cell attachment. Following the initial incubation, the culture medium was removed, and the cells were treated with varying concentrations of *Bergenia ciliata*, specifically 31.25, 62.5, 125, 250 and 500 μg/ml. The concentrations were prepared in fresh culture medium (DMEM supplemented with 1% antibiotic solution). To validate our findings, we incorporated a vehicle control group containing 0.1% dimethyl sulfoxide (the solvent for the compound) alongside an untreated control group consisting solely of the culture medium. Each concentration group and control were assessed in six replicate wells. The plates underwent incubation for an additional 24 h under identical conditions. Upon completion of the treatment period, the medium was systematically removed from each well. Subsequently, 10 µL of a 5 mg/mL MTT solution was added, prepared in sterile phosphate-buffered saline (PBS). The plates were subsequently incubated for an additional 4 h at 37 °C. Following incubation, the supernatant was aspirated, and 100 µL of dimethyl sulfoxide (DMSO) was added to each well to dissolve the formazan crystals that had formed during the process. This step is essential for precise assessment of cell viability. The plates were gently shaken for 15 min at room temperature to ensure complete dissolution. Absorbance was measured at 570 nm using a microplate reader for result analysis.

### 2.12 Animal study design

Thirty healthy male Wistar albino rats, aged 9–10 weeks and weighing approximately 180–220 g, were procured from the animal facility at Vellore Institute of Technology (VIT) in Vellore, Tamil Nadu, India. All experimental techniques involving subjects were executed in strict compliance with the norms and regulations set forth by the Committee for the Control and Supervision of Experiments on Animals (CPCSEA) of the Government of India. The investigation was documented in accordance with the ARRIVE standards version 2.0. The Institutional Animal Ethics Committee (IAEC) of VIT sanctioned the study (Approval Certificate No: VIT/IAEC/18/Dec/24/21).

The rats were housed within standardized environmental parameters, which included a regulated temperature of 27 °C ± 3 °C, a relative humidity range of 35%–50%, and a 12-h light/dark cycle. A conventional pelleted diet was given to them along with unrestricted access to water. Following a 1-week acclimatisation phase, the animals were randomly allocated into five distinct groups, with each group comprising six rats (n = 6 per group) as shown in Table 1. Dosses of the artesunate has been fixed based on the previous study (Desai et al., 2015; Keiser et al., 2006; Yang et al., 2012).

**TABLE 1:** Experimental groups and treatment details.

Groups	Treatment description
Group 1	Normal saline (Untreated control group)
Group 2	Artesunate (150 mg/kg b.w/day, p.o.) for 21 days
Group 3	Artesunate (150 mg/kg b.w/day, p.o.) + *Bergenia ciliata* root extract (200 mg/kg b.w/day, p.o.) for 21 days
Group 4	Artesunate (150 mg/kg b.w/day, p.o.) + *Bergenia ciliata* root extract (400 mg/kg b.w/day, p.o.) for 21 days
Group 5	Artesunate (150 mg/kg b.w/day, p.o.) + Silymarin (25 mg/kg b.w/day, p.o.)

### 2.13 Statistical analysis

Data are presented as mean ± standard error of the mean (SEM), taken from six independent samples (n = 6). The statistical comparisons among the five groups were performed with one-way ANOVA followed by Tukey’s *post hoc* test. A p-value 0.05 was considered statistically significant. All data analysis and data presentation were completed using GraphPad Prism v9.0, and the results were shown graphically.

### 2.14 Assesment of renal function biomarker

At the end of the 21-day experimental period, blood samples were collected from all rats via cardiac puncture. Blood was transferred into EDTA-coated tubes and centrifuged at 3,000 rpm for 15 min at 4 °C to obtain plasma. The separated plasma was stored at −20 °C until further analysis. Plasma urea, uric acid, and creatinine were estimated as indices of renal function using standard colorimetric methods.

#### 2.14.1 Estimation of plasma urea

The concentration of plasma urea was measured using the diacetyl monoxime (DAM) method developed by Wybenga, et al., (1971) (Wybenga et al., 1971). Urea in plasma reacted with diacetyl monoxime in the presence of thiosemicarbazide and ferric ions in an environment containing low pH and heat to form a pink chromogen. The pink colour formed from the complex was then measured spectrophotometrically at 530 nm for absorbance.

#### 2.14.2 Estimation of plasma uric acid

Uric acid concentration in plasma was measured using the uricase-phosphotungstate method as described by Fossati et al. (1980) (Fossati et al., 1980). Uricase converts uric acid to allantoin and H_2_O_2_. H_2_O_2_ then reacts with 4-aminoantipyrine and a chromogen in the presence of peroxidase (POD) to yield a colored quinoneimine dye, which has an intensity proportional to the concentration of uric acid in the sample. The absorbance was measured at 505 nm.

#### 2.14.3 Estimation of plasma creatinine

Plasma creatinine concentration was assessed through the alkaline picrate method. The reaction involves the formation of an orange-red coloured creatinine-picrate complex (Janovsky’s complex) as a result of the reaction between creatinine and picric acid under alkaline conditions, the absorbance of the complex was then determined via spectrophotometry at 520 nm (Slot, 1965).

### 2.15 Biochemical assesment of oxidative stress marker

Kidneys were excised, washed with ice-cold phosphate-buffered saline (PBS, pH 7.4), and homogenized in 0.1 M Tris-HCl buffer (pH 7.4) at a 1:10 (w/v) ratio using a tissue homogenizer. The homogenate was centrifuged at 3,000 rpm for 15 min at 4 °C, and the resulting supernatant was collected and used for biochemical estimations. All antioxidant enzyme activities and lipid peroxidation were assessed in kidney tissue homogenate.

#### 2.15.1 Estimation of superoxide dismutase (SOD)

The enzyme superoxide dismutase (SOD) was assessed by combining 500 µL of a tissue sample with 2 mL of 0.1 M Tris-HCl buffer (pH 8.2). The assay was initiated by the addition of 500 µL of pyrogallol to the 3 mL mixture prior to the dilution of the sample with deionized water. The absorbance was measured at 420 nm and the cessation of pyrogallol auto-oxidation was used to measure SOD activity.

#### 2.15.2 Estimation of catalase (CAT)

1 ml of phosphate buffer was mixed with 100 µl of a tissue sample to determine the activity of catalase in that sample. This mixture was then reacted with 400 µl of 0.2 m hydrogen peroxide (h2o2) for 1 min, after which the reaction was stopped by adding 2 ml of a dichromate-acetic acid reagent. After the addition of this reagent, the mixture was boiled in a boiling water bath for 10 min; then cooled, and measured for its absorbance at 620 nm.

#### 2.15.3 Estimation of reduced glutathione (GSH)

A sample analysed to determine reduced glutathione content will include 100 µL of the tissue sample, 2 mL of 5% TCA, followed by centrifugation at 5,000 rpm for 15 min; from the supernatant, 500 µL will be taken and mixed with 1 mL of DTNB reagent, then brought up to 3 mL total volume before leaving undisturbed at room temperature for 5 min prior to taking an absorbance measurement at 412 nm.

#### 2.15.4 Estimation of lipid peroxidation (MDA - TBARS method)

MDA (malondialdehyde), as an indicator of lipid peroxidation, was determined using a thiobarbituric acid reactive substances (TBARS) assay based on the procedure described by (Ohkawa et al., 1979). The reaction between MDA and thiobarbituric acid (TBA) produces a pink chromogen that can be measured spectrophotometrically at 532 nm. The results were reported as nmol MDA/mg protein.

#### 2.15.5 Estimation of glutathione peroxidase (GPx)

The method for determining Glutathione peroxidase (GPx) activity was described by Rotruck et al. (1973) (Rotruck et al., 1973) and is based on measuring the reduction in GSH (glutathione) by the enzyme while using H^2^O^2^ (hydrogen peroxide) as the substrate. The enzyme activity is expressed as µg GSH (reduced) consumed by GPx, per minute per mg of protein.

### 2.16 Histopathological analysis

After the experimental period ended, kidneys were removed from all the sacrificed rats, and perfused with ice-cold phosphate buffer (0.1 M, pH 7.4), in order to remove residual blood. The kidneys were fixed in 10% neutral buffered formalin (NBF) at room temperature for 24 h to obtain sufficient preservation of the tissues. The fixed kidneys were processed through a standardized histological protocol: dehydrated progressively through 70%, 80%, 90%, 95%, and 100% ethanol; cleared twice in xylene for 30 min; involved with paraffin wax; and embedded in paraffin wax. Blocks of paraffin-embedded tissue were sectioned with a rotary microtome at 5 µm thickness. The sections of tissue were mounted on glass slides and deparaffinized by being immersed in xylene: hydrated through descending grades of ethanol; and stained with Hematoxylin and Eosin (H&E) following standard histological protocols.

## 3 Results

### 3.1 Screening for therapeutic targets

**FIGURE 1:**
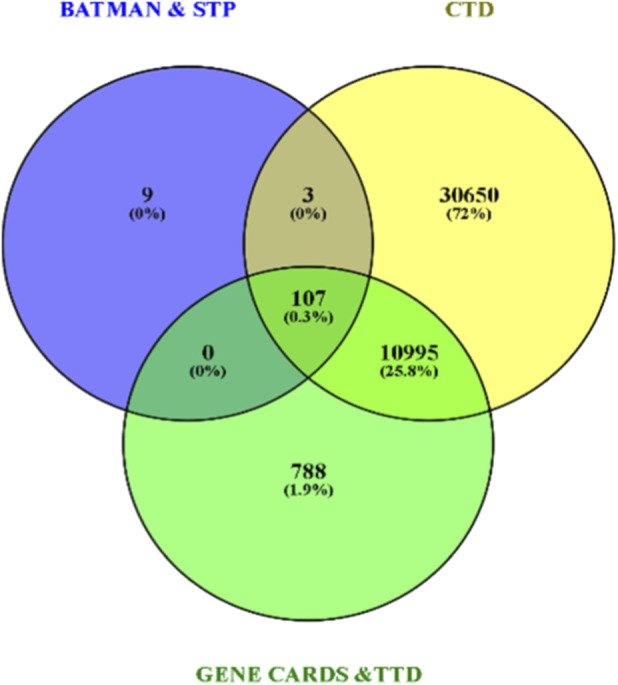
Venn diagram illustrating the overlap between Artesunate targets and disease-associated genes, emphasizing the shared regulation of nephrotoxicity-related genes.

A targeted screening approach was utilized to study the possible interactions between Artesunate and disease genes. Using a Venn diagram, the area of overlap between the targets for Artesunate and those for the disease genes was demonstrated with 107 shared targets. The coincidence provides evidence that there may be a regulatory relationship between the pathways of Artesunate and those related to the disease, especially with regard to nephrotoxicity, as illustrated in Figure 1.

### 3.2 Protein-protein interaction (PPI) network

**FIGURE 2:**
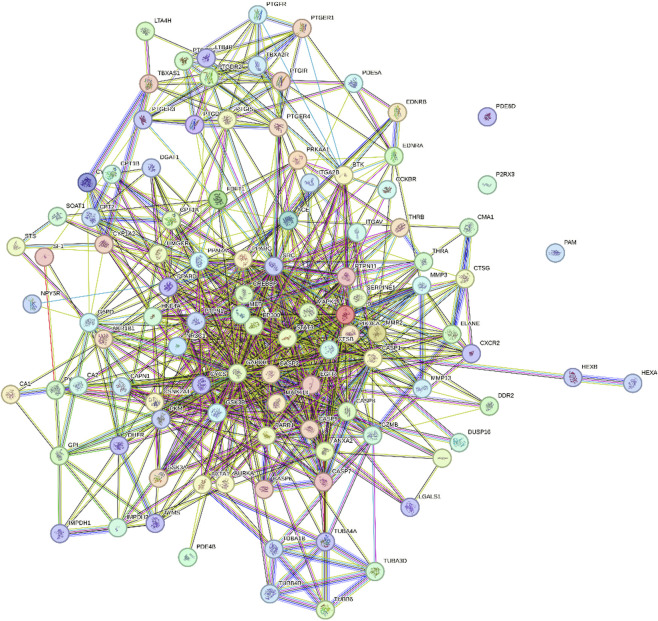
Protein–Protein Interaction (PPI) Network. The network illustrates the associations between identified protein targets, emphasizing key nodes and potential functional clusters.

A PPI network (Protein-Protein Interaction Network) was generated from the STRING database and shown in Figure 2. Each node represents a protein, while the edges indicate either predicted or established connections between proteins. This PPI network provides an overview of the molecular interactions between the targets identified and highlights potential key regulator proteins or centre hub proteins within this PPI network.

### 3.3 Hub gene identification

**FIGURE 3:**
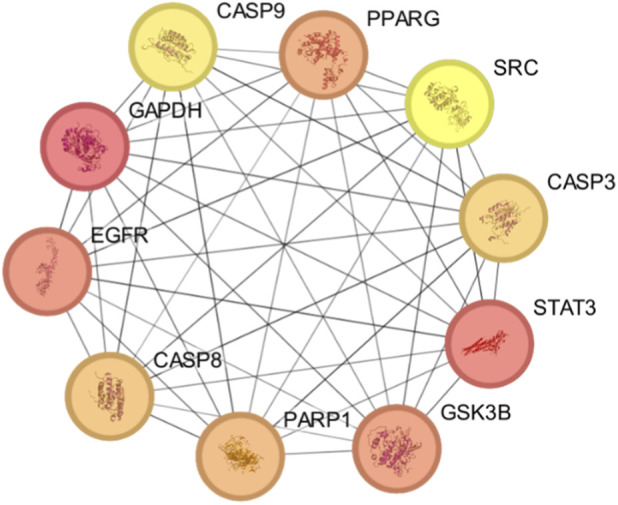
STRING network analysis showing top 10 hub genes targeted by artesunate.

Using the STRING database, construction of a protein–protein interaction (PPI) network using topological analysis identified by the CytoHubba plugin was completed (Figure 3). The identified hub genes include CASP3, CASP8, CASP9, EGFR, GAPDH, GSK3B, PARP1, PPARG, SRC and STAT3, all represented with their respective three-dimensional models. The node color intensity demonstrates the connectivity degree, where red represents high and yellow represents low degrees of node connectivity. The edges represent predicted functional protein associations; therefore, these hub target genes are closely related to the pathways for inflammatory response, cellular stress response and apoptosis. Thus, it is important to review the potential role of these hub target genes in organ toxicity due to artesunate.

### 3.4 GC-MS analysis of chloroform, methanol, ethanol, and dichloromethane (DCM) extracts from the root of *Bergenia ciliata*

The GC-MS analysis led to the identification of various bioactive compounds present in the *Bergenia ciliata* root. The chromatograms for chloroform, methanol, ethanol, and DCM revealed unique peaks corresponding to different bioactive chemicals. These compounds were identified by comparing the peak, retention duration, peak area (%), and mass spectra of the top four hits of significant phytoconstituents to known compounds classified in the National Institute of Standards and Technology (NIST) database. This spectral line indicates the exact identification of bioactive chemicals found in extracts.

The findings indicated that the chloroform extract comprised the 20 predominant compounds, the methanol extract likewise included 20 compounds, the ethanol extract had 10 compounds, and the DCM extract consisted of 6 compounds. A total of 56 unique compounds were discovered from the GC-MS analysis of the four extracts. All extracts’ spectra have been included in the Supplementary File 1. Detailed information on each compound, including their retention time (RT), molecular weight (MW), peak area percentage, and CID number, is presented in the Tables 2–5.

**TABLE 2:** Phytochemical compounds present in the chloroform extract of *Bergenia ciliata* root were identified using GC-MS analysis.

Sl. No	RT (min)	Compound name	M.W.	Formula	Peak area (%)	CID
1	16.03	OXALIC ACID, MONOAMIDE, N-PROPYL, OCTYL ESTER	243	C_13_H_25_O_3_N	15.63%	6,420,425
2	16.05	PENTANOIC ACID, 2-METHYL-, BUTYL ESTER	172	C_10_H_20_O_2_	15.63%	95,539
3	16.08	PROPANOIC ACID, 2-METHYL-	88	C_4_H_8_O_2_	15.63%	6,590
4	16.09	S-PROPYLTHIO-L-CYSTEINE	195	C_6_H_13_O_2_NS_2_	15.63%	192,762
5	17.61	1.3(Z),13-TETRADECATRIENE	192	C_14_H_24_	17.41%	5,362,691
6	17.62	1,3,11-DODECATRIENE	164	C_12_H_20_	17.41%	5,365,610
7	17.63	9,11-DODECADIEN-1-OL	182	C_12_H_22_O	17.41%	5,365,562
8	17.64	9,12-OCTADECADIEN-1-OL, (Z,Z)-	266	C_18_H_34_O	17.41%	5,365,682
9	17.67	9-AZABICYCLO [6.1.0]NONANE, 9.9′-AZOBIS-, [1.ALPHA.,8.ALPHA.,9 [E (1′R*,8′S*)]]	276	C_16_H_28_N_4_	17.48%	5,362,737
10	17.68	1,11-DODECADIENE	166	C_12_H_22_	17.48%	22,170
11	17.68	4-DODECEN-1-OL	184	C_12_H_24_O	17.48%	5,362,820
12	17.69	1,13-TETRADECADIENE	194	C_14_H_26_	17.48%	30,875
13	17.93	OXALIC ACID, MONOAMIDE, N-PROPYL, HEPTYL ESTER	229	C_12_H_23_O_3_N	17.78%	6,420,424
14	17.94	1,3-PENTANEDIOL, 4-METHYL-2-NITRO-	163	C_6_H_13_O_4_N	17.78%	537,929
15	17.94	METHYL 4-HYDROXYBUTANOATE	118	C5H10O3	17.78%	70,217
16	17.96	5-HEXENOIC ACID, METHYL ESTER	128	C_7_H_12_O_2_	17.78%	520,082
17	26.85	2-BUTANONE, 4-(ACETYLOXY)	130	C_6_H_10_O_3_	27.85%	139,100
18	26.86	3-AZONIA-5-HEXENE-1-OL, N,N-DIMETHYL-, CARBAMATE ESTER, BROMIDE	173	C_8_H_17_O_2_N_2_	27.85%	535,562
19	26.87	3-PIPERIDINOL, 1,4-DIMETHYL-, TRANS-	129	C_7_H_15_ON	27.85%	22,212,772
20	26.88	6-HYDROXY-4.4,5,6-TETRAMETHYLTETRAHYDRO-1,3-THIAZIN-2-THIONE	205	C8H_15_ONS_2_	27.85%	5,363,344

**TABLE 3:** Phytochemical compounds present in the methanol extract of *Bergenia ciliata* root were identified using GC-MS analysis.

Sl. No	RT (min)	Compound name	M.W.	Formula	Peak area (%)	CID
1	5.33	(+)-3,4-DEHYDROPROLINE AMIDE	112	C_5_H_8_ON_2_	6.84%	549,255
2	5.34	OROTYL AMIDE	155	C_5_H_5_O_3_N_3_	6.84%	248,966
3	5.35	PROLINE, 3,4-DIDEHYDRO	113	C_5_H_7_O_2_N	6.84%	25,202,244
4	5.36	3,4DEHYDRO-DL-PROLINE	113	C_5_H_7_O_2_N	6.84%	97,858
5	5.54	7-OCTEN-4-OL, 2-METHYL-6-METHYLENE-, (S)-	154	C_10_H_18_O	7.29%	85,712
6	5.54	1,3-NONADIEN-6-OL, 8-METHYL-	154	C_10_H_18_O	7.29%	5,365,762
7	5.54	PENTANOIC ACID, 2-HYDROXY-4-METHYL-, METHYL ESTER	146	C_7_H_14_O_3_	7.29%	62,908
8	5.54	1H-PYRROLE, 2,5-DIHYDRO-	69	C_4_H_7_N	7.29%	66,059
9	5.58	CYCLOBUTYL ISOPROPYLPHOSPHONOFLUORIDATE	180	C_7_H_14_O_2_FP	7.39%	549,282
10	5.58	CYCLOPENTANEMETHANOL, .ALPHA.-METHYL	114	C_7_H_14_O	7.39%	539,321
11	5.79	5-CIS-METHYL-1R,3-CIS-CYCLOHEXANEDIOL	130	C_7_H_14_O_2_	7.83%	10,964,540
12	5.79	PYRROLIDINE, 1-NITRO	116	C_4_H_8_O_2_N_2_	7.83%	77,373
13	5.96	(3R)-(+)-3-ACETAMIDOPYRROLIDINE	128	C_6_H_12_ON_2_	8.21%	7,016,129
14	5.96	(3S)-(−)-3-ACETAMIDOPYRROLIDINE	128	C_6_H_12_ON_2_	8.21%	7,021,471
15	5.12	CYCLOBUTANOL	72	C_4_H_8_O	6.37%	76,218
16	5.12	GLYCIDOL	74	C_3_H_6_O_2_	6.37%	11,164
17	5.12	OXIRANEMETHANOL, (S)-	74	C_3_H_6_O_2_	6.37%	6,973,630
18	6.12	ACETAMIDE, N-METHYL-N-(2-PROPYNYL)-	111	C_6_H_9_ON	8.56%	29,033
19	6.12	N-(1,1-DIMETHYL-PROP-2-YNYL)-ACETAMIDE	125	C_7_H_11_ON	8.56%	1,263,582
20	6.12	3,4DEHYDRO-DL-PROLINE	113	C_5_H_7_O_2_N	8.56%	97,858

**TABLE 4:** Phytochemical compounds present in the ethanol extract of *Bergenia ciliata* root were identified using GC-MS analysis.

Sl. No	RT (min)	Compound name	M.W.	Formula	Peak area (%)	CID
1	5.31	4-PENTEN-1-YL ACETATE	128	C_7_H_12_O_2_	8.61%	74,096
2	5.31	N-(1,1-DIMETHYL-PROP-2-YNYL)-ACETAMIDE	125	C_7_H_11_ON	8.61%	1,263,582
3	5.39	1H-PYRROLE, 2,5-DIHYDRO-	69	C_4_H_7_N	8.83%	66,059
4	5.39	PROLINE, 3,4-DIDEHYDRO-	113	C_5_H_7_O_2_N	8.83%	97,858
5	5.39	3,4DEHYDRO-DL-PROLINE	113	C_5_H_7_O_2_N	8.83%	568,381
6	5.86	ACETAMIDE, N-METHYL-N-(2-PROPYNYL)	111	C_6_H_9_ON	10.14%	29,033

**TABLE 5:** Phytochemical compounds present in the DCM extract of *Bergenia ciliata* root were identified using GC-MS analysis.

Sl. No	RT (min)	Compound name	M.W.	Formula	Peak area (%)	CID
1	5.25	PROLINE, 3,4-DIDEHYDRO-	113	C_5_H_7_O_2_N	15.29%	94,284
2	5.25	CYCLOPENTANE, (1-METHYLETHYL)-	112	C_8_H_16_	15.29%	19,751
3	5.25	4-PENTEN-1-YL ACETATE	128	C_7_H_12_O_2_	15.29%	74,096
4	5.25	4-HEPTYN-2-OL	112	C_7_H_12_O	15.29%	89,225
5	5.25	OROTYL HYDRAZIDE	170	C_5_H_6_O_3_N_4_	15.29%	72,690
6	5.25	CYCLOBUTYL ISOPROPYLPHOSPHONOFLUORIDATE	180	C_7_H_14_O_2_FP	15.29%	549,282
7	5.25	3-DIMETHYLAMINOMETHYLENE-5,5-DIMETHYLFURAN-2,4-DIONE	183	C_9_H_13_O_3_N	15.29%	5,362,962
8	11.29	5-ACETOXYMETHYL-2-FURALDEHYDE	168	C_8_H_8_O_4_	45.58%	66,349
9	11.29	DIMETHYLMUCONIC ACID	170	C_8_H_10_O_4_	45.58%	5,369,045
10	11.29	2-CYCLOPENTEN-1-ONE, 5-HYDROXY-2,3-DIMETHYL	126	C_7_H_10_O_2_	45.58%	534,047

LC-MS analysis of the methanolic extract of *Bergenia ciliata*, as reported in the referenced study, revealed the presence of seven major compounds 7-Hydroxymitragynine, Pyridoxamine 5′-phosphate, Bergenin, Ajmaline, Saikosaponin b2, Chelidonine, and Quercetin-4′-O-glucoside (Das et al., 2025). GC-MS and LC-MS are complementary analytical techniques that detect distinct classes of phytochemical compounds. GC-MS is optimized for volatile and semi-volatile low-molecular-weight compounds, whereas LC-MS excels at identifying non-volatile, thermally labile, and high-molecular-weight compounds such as poly-phenolic glycosides and alkaloids. Consequently, the compound profiles obtained from each technique are inherently different and are not expected to overlap. Together, they provide a more comprehensive phytochemical characterization of *B. Ciliata* root extract. The LC-MS-derived bioactives reported from the reference study (Table 6), particularly Rutin, were included in the *in silico* analyses owing to their well-established pharmacological relevance and documented presence in *B. Ciliata*.

**TABLE 6:** List of phytochemical compounds detected in *Bergenia ciliata* according to the reference study.

Sl. No	Phytochemicals	Chemical formula	CID	Citation
1	Bergenin	C_14_H_16_O_9_·H_2_O	66,065	Singh et al. (2017)
2	Tannic acid	C_76_H_52_O_46_	16,129,778	Singh et al. (2017)
3	Gallic acid	C_6_H_2_(OH)_3_COOH	370	Singh et al. (2017)
4	Catechin	C_15_H_14_O_6_	9,064	Chauhan et al. (2013)
5	Gallicin	C_8_H_8_O_5_	101,616,642	Pokhrel et al. (2014)
6	Arbutin	C_12_H_16_O_7_	440,936	Dharmender et al. (2010)
7	Hexanal	C_6_H_12_O	6,184	Vinesh Kumar and Devendra Tyagi (2013)
8	Quercetin Rhamnodiglucoside	C_33_H_40_O_21_	157,009,882	Aziz et al. (2023)
9	Rutin	C_27_H_30_O_16_	5,280,805	Zafar et al. (2019); Kour et al. (2021)
10	Morin	C_15_H_10_O_7_	5,281,670	Kour et al. (2021)

### 3.5 High-throughput virtual screening (HTVS) of phytochemical compounds derived from the crude extract of *Bergenia ciliata*

Seventy-nine bioactive phytochemical compounds derived from *Bergenia ciliata* were prepared using the LigPrep module of Schrödinger Suite 2025. This preparation step involved the generation of low-energy, three-dimensional structures with optimized geometries and appropriate protonation states. Following virtual screening through molecular docking, the top 4 candidate compounds were selected based on their highest binding affinities (docking scores) and favourable binding free energies, as determined by the MM-GBSA (Molecular Mechanics/Generalized Born Surface Area) method. The detailed results are presented in Table 7. The top 4 hits were selected for further analysis based on their strong binding interaction, demonstrating superior performance compared to the positive control.

**TABLE 7:** List of the top 4 bioactive compounds with their highest docking score and MMGBSA analysis against the PPARγ protein.

S. no.	Compound ID	IUPAC name	Docking score (Kcal/mol)	MMGBSA (Kcal/mol)
1	5,280,805	2-(3,4-dihydroxyphenyl)-5,7-dihydroxy-3-[(2S,3R,4S,5S,6R)-3,4,5-trihydroxy-6-[[(2R,3R,4R,5R,6S)-3,4,5-trihydroxy-6-methyloxan-2-yl]oxymethyl]oxan-2-yl]oxychromen-4-one	−10.307	−71.66
2	12,442,954	3,5,7-trihydroxy-2-[3-hydroxy-4-[3,4,5-trihydroxy-6-(hydroxymethyl)oxan-2-yl]oxyphenyl]chromen-4-one	−8.358	−56.85
3	440,936	(2R,3S,4S,5R,6S)-2-(hydroxymethyl)-6-(4-hydroxyphenoxy)oxane-3,4,5-triol	−8.313	−64.19
4	66,065	(2R,3S,4S,4aR,10bS)-3.4,8,10-tetrahydroxy-2-(hydroxymethyl)-9-methoxy-3.4,4a,10b-tetrahydro-2H-pyrano [3,2-c]isochromen-6-one	−7.733	−45.99
Positive control				
5	5,213	3,5,7-trihydroxy-2-[3-(4-hydroxy-3-methoxyphenyl)-2-(hydroxymethyl)-2,3-dihydro-1,4-benzodioxin-6-yl]-2,3-dihydrochromen-4-one	−7.7	−40.23

### 3.6 Evaluation of the pharmacokinetic and pharmacodynamic characteristics of the identified hit compounds

We did a complete study of the absorption, distribution, metabolism, and excretion (ADME) properties of the chosen substances, comparing the results to known reference ranges (Beaumont et al., 2014). ADME profiling was carried out using the QikProp v5.9 tool, which enabled the assessment of several key pharmacokinetic parameters.

One of the most important variables examined in this study was QPlogHERG, which estimates the likelihood of a chemical blocking HERG K^+^ channels (commonly known as a predictor of heart rhythm disorders). The identification of chemicals that produce low QPlogHERG values is significant with respect to decreasing the risk for heart damage and improving the overall safety profile of drugs. We additionally examined QPlogS (water solubility), permeability and other related measures as a means of determining the ease at which compounds will penetrate both the body and CNS.

Our evaluation provided evidence that the lead drugs exhibit favorable characteristics in their pharmacokinetic and pharmacodynamic profiles, as well as other important attributes, including molecular weight and the Solvent Accessible Surface Area (SASA) (e.g., including FOSA and PISA metrics). The volume of each drug is located in an acceptable range when presented in Table 8. The majority of these attributes are related to the SASA of the chemical compound by quantifying its interaction with the environment via its solvent and hydrophobic/hydrophilic character and the specific carbon-hydrogen bonds that exist within the molecule (PISA). Collectively, these features provide essential information regarding the potential for the drug to be developed into a therapeutic agent (Srivastava et al., 2024).

**TABLE 8:** ADME analysis of the top 4 selected compounds.

S.No	Compound name	MW (130–725)	SASA (300–1,000)	FOSA (0–750)	PISA (0–450)	Volume (500–2000)	QPlogS (−6.5 to 0.5)	QPlogHERG (above −5.0)	QPlogKp (−8.0 to −0.1)	QPlogKhsa (−1.5 to 1.5)
1	5,280,805	610.52	785.66	174.11	176.0	1,554.46	−2.13	−5.18	−7.48	−1.26
2	12,442,954	464.38	712.41	116.65	195.68	1,270.53	−3.02	−5.76	−7.14	−0.88
3	440,936	272.25	491.97	115.68	148.94	828.45	−1.53	−4.393	−4.41	−1.13
4	66,065	328.27	511.93	201.26	53.79	902.16	−1.72	−3.698	−5.39	−0.33

### 3.7 Molecular interaction study of the top compounds against the PPARγ receptor protein

**FIGURE 4:**
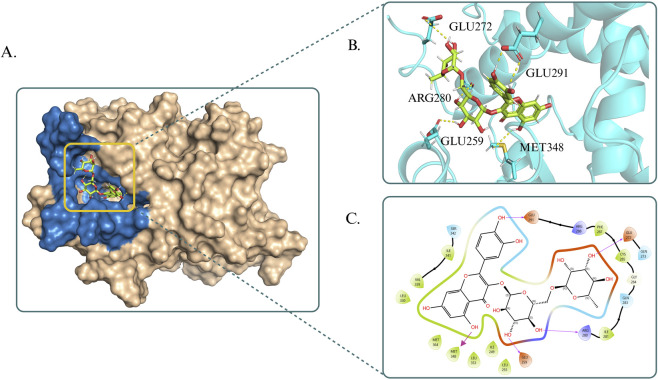
Illustration of the 3D and 2D interaction between PPARγ and CID5280805. **(A)** Surface representation shows the ligand binding mode within the active site pocket (highlighted in blue) of the PPARγ protein. **(B)** 3D representation of molecular interactions between the protein and ligand, highlighting key bond formations within the binding site. **(C)** Protein-Ligand 2D interaction map.

The top 4 candidate compounds were screened after a comprehensive evaluation of multiple parameters, including docking scores, MM-GBSA binding energies, pharmacokinetic profiles, and drug-likeness characteristics. Among them, the compound’s highest docking score and high binding affinity (5,280,805), along with PPARγ proteins, are selected for detailed molecular interaction studies. Their molecular interaction has been shown in Figure 4. The 3D protein-ligand interactions were visualized using the educational version of PyMOL (Schrodinger, 2015), while 2D interaction analyses were conducted using Schrödinger 2025.

#### 3.7.1 Molecular interaction of CID5280805 - PPARγ receptor protein

The results of the study showed that CID5280805 has the highest docking score and binding affinity towards the binding site pocket of the PPARγ protein through conventional hydrogen bonding with ARG280, GLU259, MET348, GLU291, GLU272, and forms a carbon-hydrogen bond with SER342. Figure 4 displays a 3D representation of the interaction between CID5280805 and PPARγ protein.

### 3.8 Molecular dynamics simulation

Molecular Dynamics Simulations (MDS) have been performed for 200 ns on the protein complex of PPARγ-CID 5280805 to understood the stability of the protein complex as it functions in a biological environment susceptible to change. The trajectory files of each molecular dynamics simulation were examined for the behaviour of the protein with respect to the ligand. Areas of focus were several important parameters to evaluate the behaviour of the protein complexes: 1) The Root Mean Square Deviation (RMSD) is used to quantify the displacement of the Cα atoms of PPARγ over time from their position at time zero; and 2) The Root Mean Square Fluctuation (RMSF) provides insight into the variance of individual amino acids of PPARγ from their starting conformation. Prior to performing the Molecular Dynamics Simulations (MDS), the systems were fully prepared, including energy minimization of the models, and equilibrated under NPT conditions (300K and 1.01 bar). The MDS was conducted for 200 ns and data collection occurred every 200 ps resulting in 2000 frames of data.

#### 3.8.1 RMSD, RMSF, and hydrogen bond plot analysis of the protein-ligand complex

**FIGURE 5:**
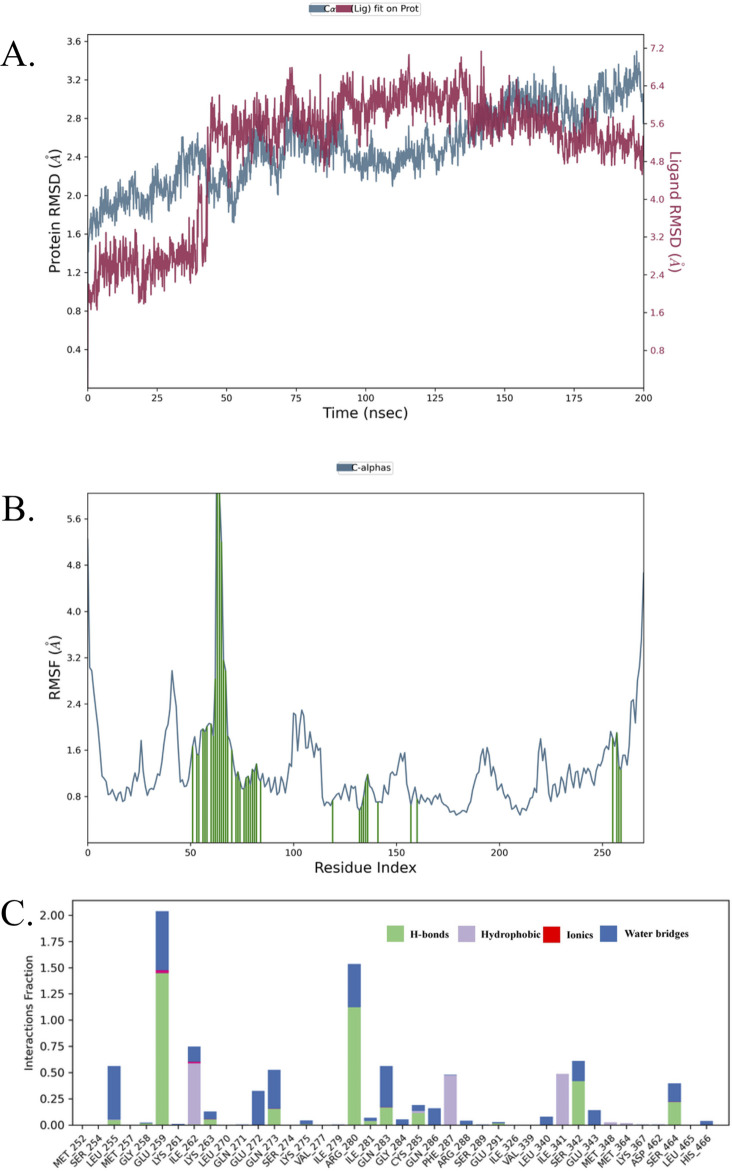
Molecular dynamics simulation diagram of PPARγ-CID5280805, representing **(A)** RMSD, **(B)** RMSF, **(C)** Protein-ligand interaction.

The structural stability of the PPARγ-Rutin complex was assessed by monitoring the root mean square deviation (RMSD) of the protein Cα backbone and the ligand over 200 ns as shown in Figure 5A. The PPARγ backbone RMSD increased gradually from ∼1.0 Å at the onset of the simulation, stabilizing at approximately 2.5–3.2 Å after ∼75 ns, indicating that the receptor underwent initial conformational relaxation before reaching a stable equilibrated state. This behaviour is consistent with the known flexibility of PPARγ′s ligand-binding domain (LBD), which undergoes adaptive conformational changes upon ligand accommodation. The RMSD of Rutin (fit on protein) fluctuated between ∼2.0–6.5 Å throughout the trajectory. While greater than the protein backbone deviation, these values reflect the dynamic repositioning of Rutin within the large, hydrophobic PPARγ ligand-binding pocket - a cavity known to accommodate structurally diverse ligands through induced-fit binding. Importantly, the ligand RMSD did not exceed ∼7 Å, confirming that Rutin remained stably bound within the active site without dissociation over the entire 200 ns simulation.

RMSF analysis of PPARγ Cα atoms revealed the flexibility profile of the receptor in the presence of Rutin. Ligand-contacting residues are highlighted in green as shown in Figure 5B. The majority of Rutin-interacting residues displayed RMSF values below 1.5 Å, reflecting a well-ordered and rigid binding interface. This rigidity is characteristic of the PPARγ LBD core, which maintains structural integrity upon binding of agonistic ligands. The prominent flexibility peak observed around residues 55–60 (∼5.8 Å) corresponds to a surface-exposed flexible loop region distal from the ligand-binding pocket, and is unlikely to influence ligand binding. Secondary flexibility peaks around residues 80–110 and 130–145 correspond to loop regions flanking the LBD. The C-terminal region (∼residues 260–270) also showed elevated flexibility, consistent with the inherent mobility of the activation function-2 (AF-2) helix region of PPARγ, which is sensitive to ligand-induced conformational changes.

LYS 261 and ILE 280 were identified as the primary interacting residues, with interaction fractions of ∼2.0 and ∼1.55, respectively. LYS 261 formed persistent hydrogen bonds with Rutin’s hydroxyl groupsas shown in Figure 5C, while ILE 280, LEU 254, ILE 262, and LEU 263 provided hydrophobic stabilization of the flavonoid scaffold. Additional polar interactions with GLN 272 and GLU 273 stabilized the rutinose sugar moiety, and water-bridged contacts at VAL 339 and SER 341 further supported binding.

### 3.9 Cell viability assay

**FIGURE 6:**
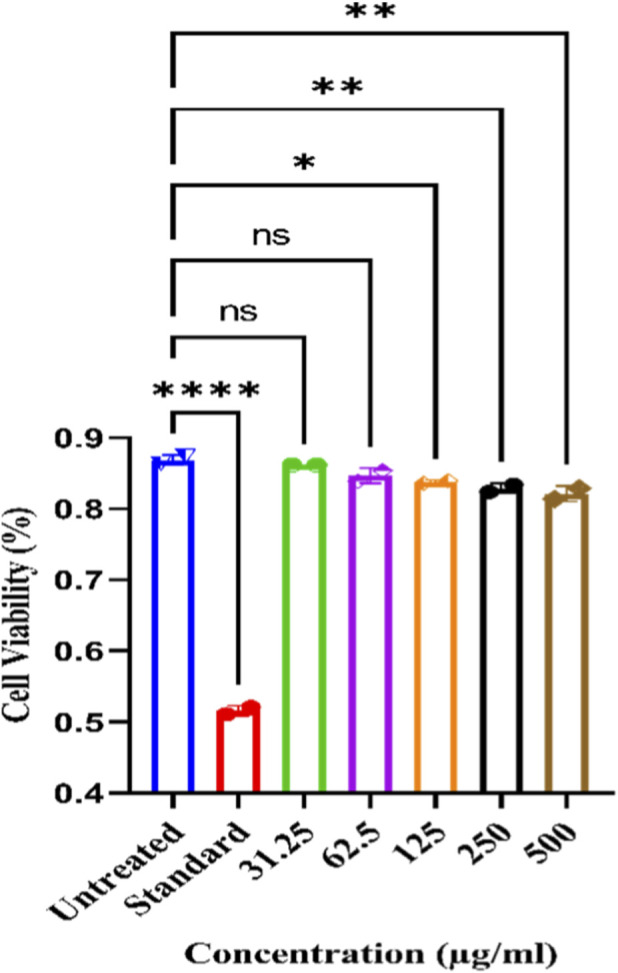
Bar graphs represent the cytotoxic effects of Bergenia ciliata root extract at varying concentrations (31.25, 62.5, 125, 250, and 500 μg/mL) assessed using the MTT assay in the HEK293 human embryonic kidney cell line.

Cytotoxicity of *Bergenia ciliata* was studied on HEK293 cells using the MTT assay at a range of concentrations (31.25–500 μg/mL). As shown in Figure 6, the untreated control group showed ∼100% viability, showing that cells are growing normally and confirming that the assay is valid. The standard-treated group showed marked decreases in cell viability with ∼58–60% of cells still viable compared to the control group, thus indicating significant cytotoxicity of the standard treatment. *(Bergenia ciliata)* treatment showed that cell viability was maintained at a high level across all concentration points tested in theory. For example, at 31.25 μg/mL, cells showed ∼99% viability, while continuing to decline minimally and without significance as concentrations increased (e.g., ∼94–95% at 500 μg/mL). Importantly, cell viability was always >90% regardless of concentration, supporting that SJ01 had negligible cytotoxicity. No clear cytotoxic trend was noted for *Bergenia Cilita* in a dose-dependent manner suggesting there is a large safety margin. Furthermore, *Bergenia Cilita* -cell viability when compared to the standard treatment was substantially greater than the standard treatment group indicating that *Bergenia Cilita* demonstrates superior cytocompatibility with HEK293 cells. MTT assay results are provided in Supplementary File 2 for reference.

### 3.10 Assessment of animal body weight

**FIGURE 7:**
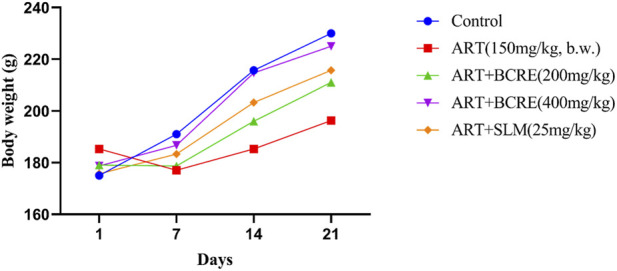
Effect of ART and combination treatments on body weight of Wistar albino rats over 21 days.

Weight measurements were taken at intervals - day 1, then again on days 7, 14, and 21 - to monitor general health and possible toxic responses in Wistar albino rats throughout treatment, with results shown in Figure 7. While the control animals steadily gained mass, reflecting typical development and absence of any toxic substance, those given only artesunate (ART), marked by the red line, lost weight progressively - a sign that the compound may trigger widespread physiological stress. Instead of declining like the ART cohort, subjects treated additionally with *Bergenia ciliata* (Groups 3 and 4) regained near-normal growth patterns. Among these, the higher dose produced the strongest protective effect, nearly preserving expected weight increases. Though affected early, this subgroup rebounded faster than others exposed solely to ART. Still, animals given both silymarin and ART had milder effects - some return of lost weight was seen. This hints that *Bergenia ciliata* could protect the body from damage caused by ART, especially when used in larger amounts.

### 3.11 Effects of artesunate on kidney function tests

**FIGURE 8:**
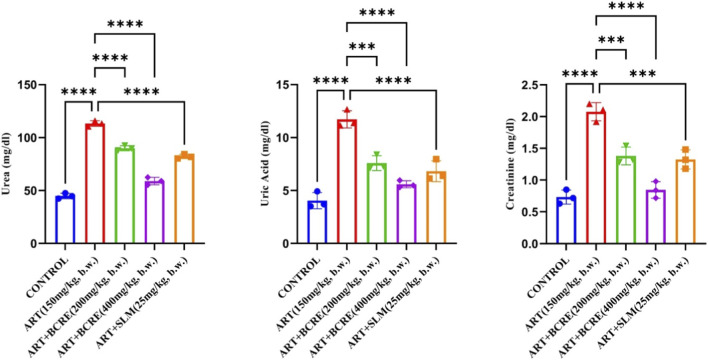
Effect of *Bergenia ciliata* root extract (BCRE) and Silymarin (SLM) on serum kidney function biomarkers in ART-induced nephrotoxicity in rats. Bar graphs display the serum levels of Urea, Uric Acid, and Creatinine across five experimental groups: Control, ART (150 mg/kg b.w.), ART + BCRE (200 mg/kg b.w.), ART + BCRE (400 mg/kg b.w.), and ART + SLM (25 mg/kg b.w.). ART treatment significantly elevated all three biomarkers compared to control, indicating nephrotoxicity. Co-treatment with BCRE, especially at 400 mg/kg, markedly reduced biomarker levels, suggesting a protective effect. Silymarin also showed moderate nephroprotection. Data are presented as mean ± SEM (n = 6). Statistical significance was determined by one-way ANOVA followed by multiple comparison tests: *p* < 0.05.

The administration of high dose of ART (150 mg/kg) led to a significant increase (p < 0.0001) in serum urea, uric acid and creatinine levels as compared to the control group indicating deterioration of renal function. Monotherapy with ART led to the highest levels of urea in the serum as compared to the other treatment groups. However, groups that were administered BCRE at the doses of 200 and 400 mg/kg exhibited a significant reduction in serum urea levels compared to the ART treated group. Similar results were obtained for uric acid levels (Figure 8). ART treatment led to significantly increased levels of uric acid in the serum as compared to the control group. However, groups that were administered BCRE exhibited a significant reduction in serum uric acid levels compared to the ART treated group. Additionally, levels of creatinine were also significantly reduced by the addition of BCRE to the ART treatment regimen. Treatment with silymarin at the dose of 25 mg/kg (SLM) led to a moderate reduction in urea, uric acid, and creatinine levels compared to the ART treated group. However, the extent of reduction in these parameters with silymarin treatment was less pronounced than that seen with BCRE. These results, therefore, indicate that BCRE has enhanced protective effects on the kidneys compared to silymarin and ART monotherapy alone.

### 3.12 Kidney antioxidant enzyme and lipid peroxidation

**FIGURE 9:**
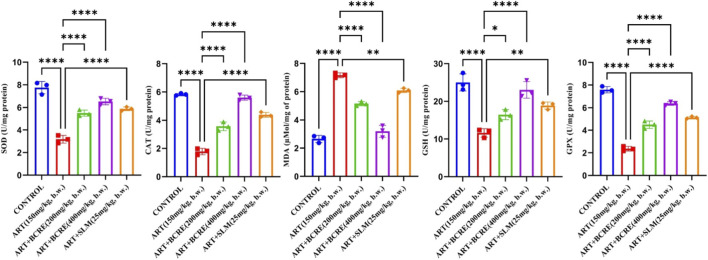
The bar graphs depict the impact of *Bergenia ciliata* root extract (BCRE) and Silymarin (SLM) on oxidative stress markers in ART-induced nephrotoxicity. The parameters shown include levels of SOD, CAT, MDA, GSH, and GPx across various treatment groups: Control, ART, ART + BCRE (200 mg/kg b.w.), ART + BCRE (400 mg/kg b.w.), and ART + SLM (25 mg/kg b.w.). Data are expressed as the mean ± standard error of the mean (SEM) for six animals per group (n = 6). Statistical comparisons were performed using one-way ANOVA, followed by Tukey’s *post hoc* test. Significance value (p < 0.05) is compared with the toxic group.

The results showed a significant effect of treatments on the activity of renal antioxidant enzymes and on lipid peroxidation. ART treatment significantly reduced the activity of SOD, CAT, GSH, and GPx enzymes as hown in Figure 9. Levels of MDA were significantly higher in the ART-treated group than in the control group. Co-administration of BCRE at both low and high doses restored the activity of antioxidant enzymes and reduced the levels of MDA in the renal tissues in a dose-dependent manner. SLM treatment significantly improved the levels of antioxidant enzymes and MDA, though to a lesser extent than BCRE at the higher dose.

### 3.13 Histopathological evaluation

**FIGURE 10:**
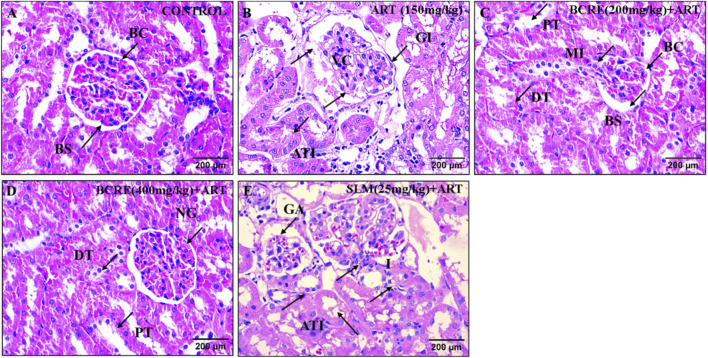
Histopathological changes in kidney sections of experimental groups stained with Hematoxylin and Eosin (H&E) (200 μm scale bar). **(A)** Control group: Preserved renal architecture with normal glomerulus (G), Bowman’s capsule (BC), and Bowman’s space (BS). **(B)** ART (150 mg/kg): Glomerular injury (GI) with widened Bowman’s space, vacuolated cytoplasm (VC), and signs of acute tubular injury (ATI). **(C)** BCRE (200 mg/kg) + ART: Partially restored glomerular structure with mild interstitial inflammation (MI), proximal tubules (PT), and distal tubules (DT) showing reduced degeneration. **(D)** BCRE (400 mg/kg) + ART: Nearly normal glomerulus (NG) with intact distal (DT) and proximal tubules (PT). **(E)** SLM (25 mg/kg) + ART: Moderate improvement in glomerular architecture (GA) with reduced acute tubular injury (ATI) and inflammation (I).

Kidney sections from group 1 had normal renal architecture and normal looking glomeruli and tubules. No evidence of acute tubular injury, interstitial nephritis, glomerulonephritis, abnormal deposits or fibrotic changes were found. In the group 2 kidney sections, the glomeruli showed evidence of atrophy with widening of the lumens of the tubular epithelium and the presence of tubular dilation with flattening of the tubular epithelial cells (Figure 10). There was mild chronic inflammation with early fibrosis, indicated by interstitial changes. There is no significant inflammatory infiltrates, which is good evidence for a chronic degenerative process rather than an acute infection. There were mild degenerative changes in group 3 (focal cytoplasmic vacuolation and minimal tubular dilatation). Group 4 kidney sections showed a relatively well preserved cortical architecture with no significant degeneration and less vacuolation, suggesting that there is less toxin-induced injury. In group 5 kidney sections, there was significant degenerative changes including severe cytoplasmic vacuolation, swelling of the tubular epithelial cells and tubular lumen dilatation. The glomeruli were slightly congested and were otherwise fairly normal. The scale was generated using ImageJ software (Schneider et al., 2012).

## 4 Discussion

Artesunate represents one of the most important antimalarial drugs in modern medicine, serving as a cornerstone of artemisinin-based combination therapies (ACTs) for treating malaria worldwide (Lai et al., 2025). The primary mechanism of artesunate begins with its activation by haemoglobin-derived ferrous heme or non-heme ferrous ions within infected erythrocytes (Zhan et al., 2023; Jena and Prince, 2025). This iron-catalysed reaction cleaves the endo-peroxide bridge, generating highly reactive carbon-centered radicals and other reactive oxygen species (ROS) (Heller and Roepe, 2019).

Demand for phyto-therapeutic agents to treat various diseases is always increasing and is being heavily explored for their use in various health issues management (Rausch et al., 2024). Many botanicals have shown significant effectiveness in reducing chemically mediated oxidative stress of liver and kidney diseases (Jena and Prince, 2025). Pharmacological agents derived from plants are widely used in different countries, mostly as part of the traditional medicine system (Nasim et al., 2022). However, in order to make use of these herbal formulations effectively and wisely it is important to scientifically test their effectiveness. The current research was designed to evaluate the ameliorative effect of *B. Ciliata* root extract in the prevention of renal damage caused by ART in rats both *in silico*, *in-vitro*, and *in-vivo*. In rats, *B. Ciliata* extract was effective in reducing the serum levels of urea, uric acid and creatinine in a dose-dependent manner when co-administered with high dose ART. Traditionally *B. Ciliata* has been known to be effective in expelling kidney stones and as an anti-malarial. Among the different combination therapies tested, the combination of *B. Ciliata* extract with ART showed the most remarkable reno-protective effect. Based on the results obtained from this study, it is concluded that *B. Ciliata* extract (400 mg/kg) can effectively reduce the nephrotoxicity induced by ART, especially at high doses of ART.

We employed the network pharmacology method to screen the most important 10 proteins that are associated with ART-induced kidney toxicity. Of these, the importance of PPAR-γ in renal injury made it the primary target. ART was shown to reduce the activation of PPAR-γ and thus the renal reparative mechanisms (Jang, 2016; Elshazly and Soliman, 2019). Based on the results obtained from *in silico* analysis, we found that rutin (Zafar et al., 2019). The main bioactive compound in the crude extract of *B. Ciliata* displayed maximum binding affinity towards PPARγ which indicates its possible activation of this receptor. Molecular dynamics simulation (MDS) over 200 ns confirmed dynamic stability of the PPARγ-Rutin complex. The protein backbone RMSD remained stable (2.4–3.2 Å) throughout, confirming structural integrity of PPARγ. The ligand RMSD showed a transient elevation (5.5–6.5 Å) between 40–150 ns, attributable to the conformational flexibility of Rutin’s glycosylated scaffold (MW: 610.52 Da), before stabilizing at ∼4.5 Å -consistent with convergence to a thermodynamically favourable binding pose. Sustained hydrogen bond interactions with GLU259 and ARG280 throughout the simulation confirmed that despite transient RMSD fluctuations, Rutin maintained essential polar contacts necessary for PPARγ activation. Collectively, the MDS data support that Rutin engages PPARγ in a dynamically stable and productive manner, consistent with its role as a potential PPARγ agonist capable of reversing ART-induced suppression of renal repair pathways.

The extract demonstrated protective properties against nephrotoxicity by showing similar results from *in-vitro* testing in HEK293 cells to the results obtained from examining group 4 wistar albino rats. These findings provide additional evidence that *Bergenia ciliata* has potential as a therapeutic tool to combat ART-induced nephrotoxicity by providing a complementary mechanism of action as well as establishing its safety profile. It is important to note that the artesunate dose (150 mg/kg/day for 21 days) administered for this investigation was a supratherapeutic/induction dose (Keiser et al., 2006; Yang et al., 2012). A key limitation of this study is the absence of *in vivo* PPARγ protein expression analysis in kidney tissue.

## 5 Conclusion

Oral administration of *B. Ciliata* root extract in a dose-dependent manner significantly reduced elevated plasma renal biomarkers (urea, uric acid, and creatinine) in Wistar albino rats subjected to high-dose ART treatment. Co-administered *B. Ciliata* extract with ART at higher doses effectively normalized ART-induced nephrotoxicity. However, the proposed PPARγ-mediated nephroprotective mechanism of *B. Ciliata*, while strongly supported by integrated *in silico* evidence, requires further *in vivo* validation through PPARγ protein expression analysis (Western blotting/IHC) and downstream signalling markers (Nrf2, NF-κB, TNF-α) in kidney tissue to conclusively establish the molecular basis of the observed protective effects. Future studies incorporating these molecular endpoints will conclusively establish the mechanistic basis of *B. Ciliata* nephroprotection and support its clinical translation.

## Data Availability

The original contributions presented in the study are included in the article/Supplementary Material, further inquiries can be directed to the corresponding author.
