# Supplementary figures and images for: Protective role of Bergenia ciliata in artesunate-induced nephrotoxicity: a translational pharmacology study integrating in-silico and experimental evidence
Source: Front Pharmacol. 2026 Jun 24;17:1862707. doi: 10.3389/fphar.2026.1862707 (PMC13341887; doi:10.3389/fphar.2026.1862707)

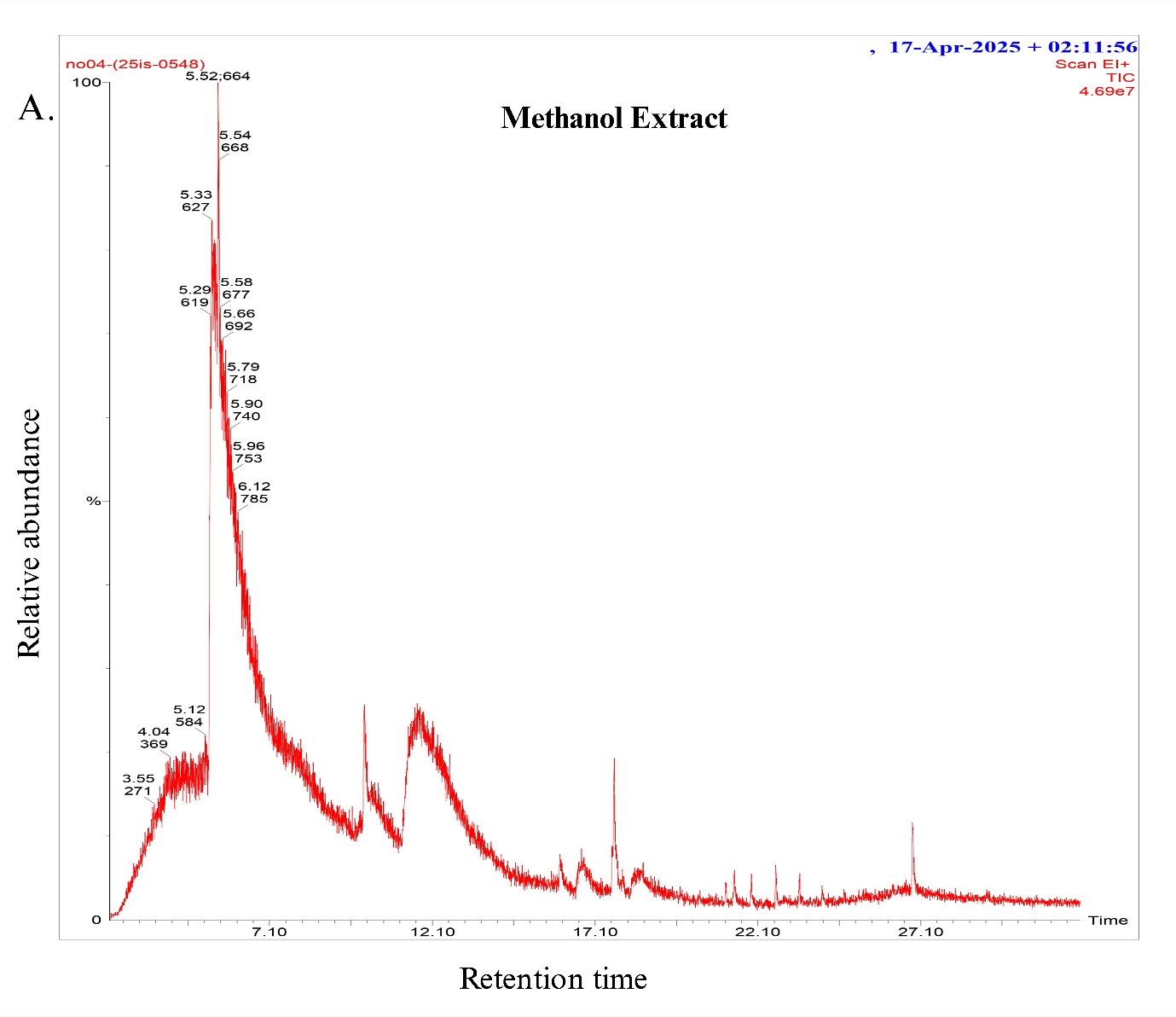

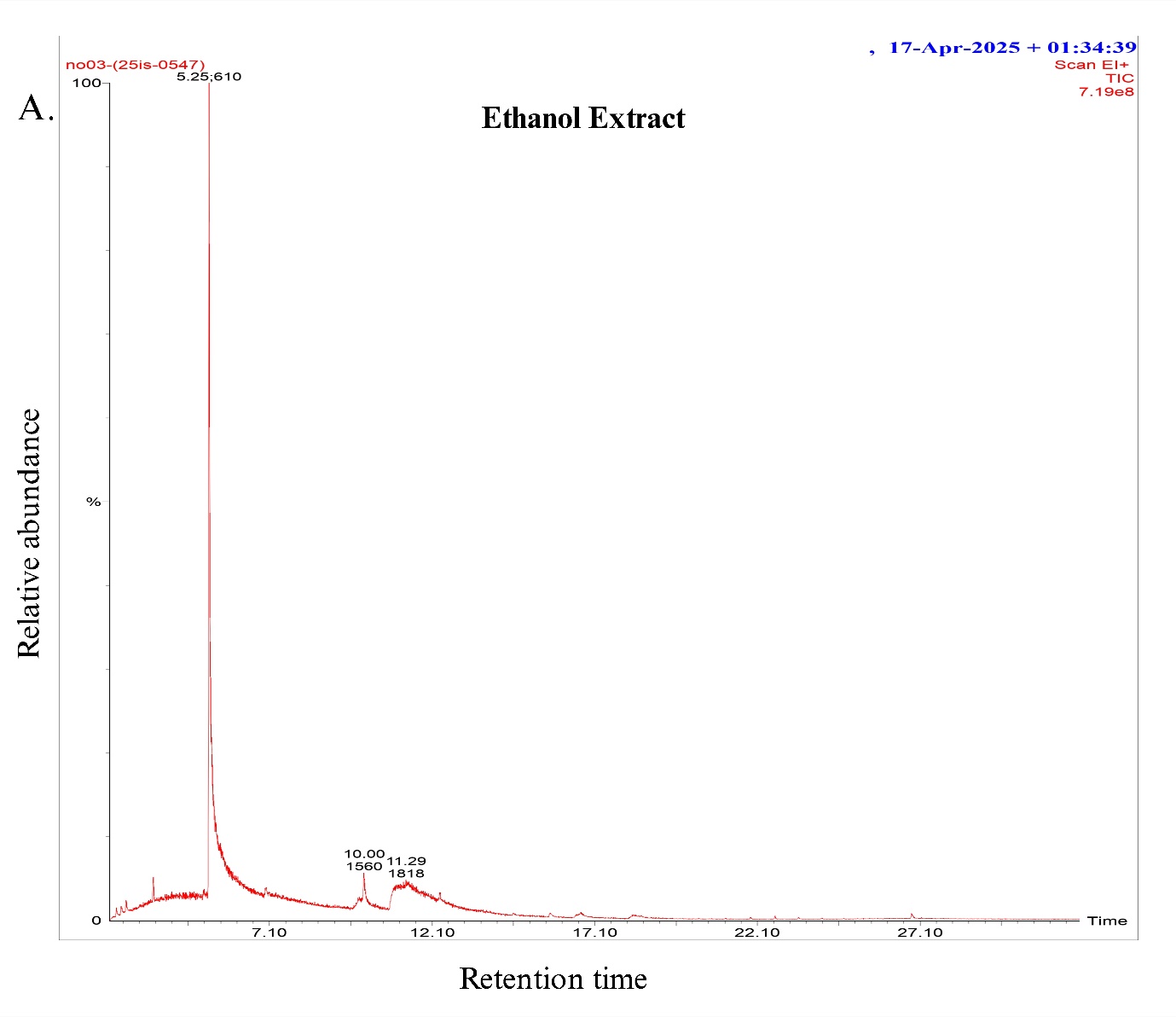

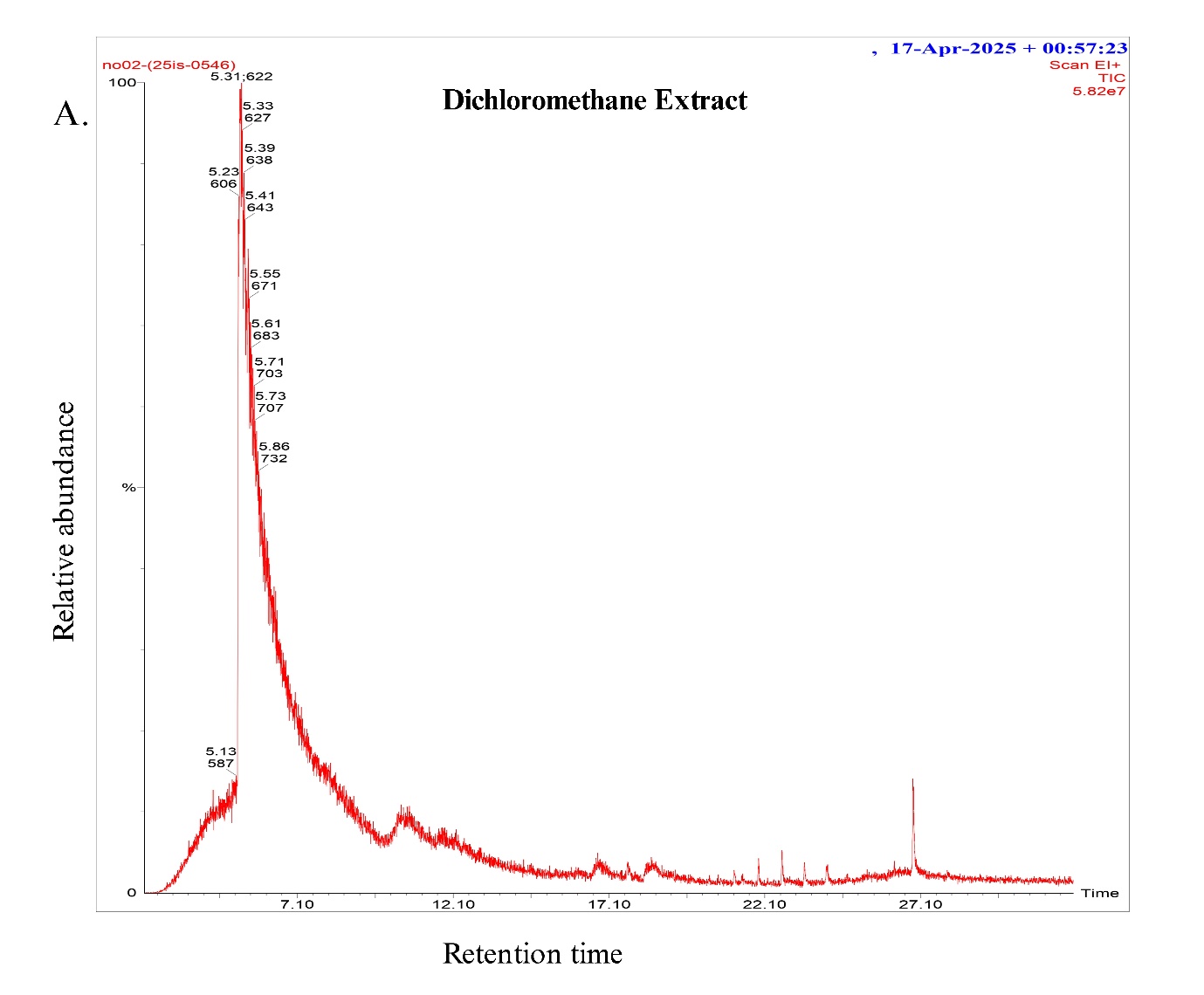

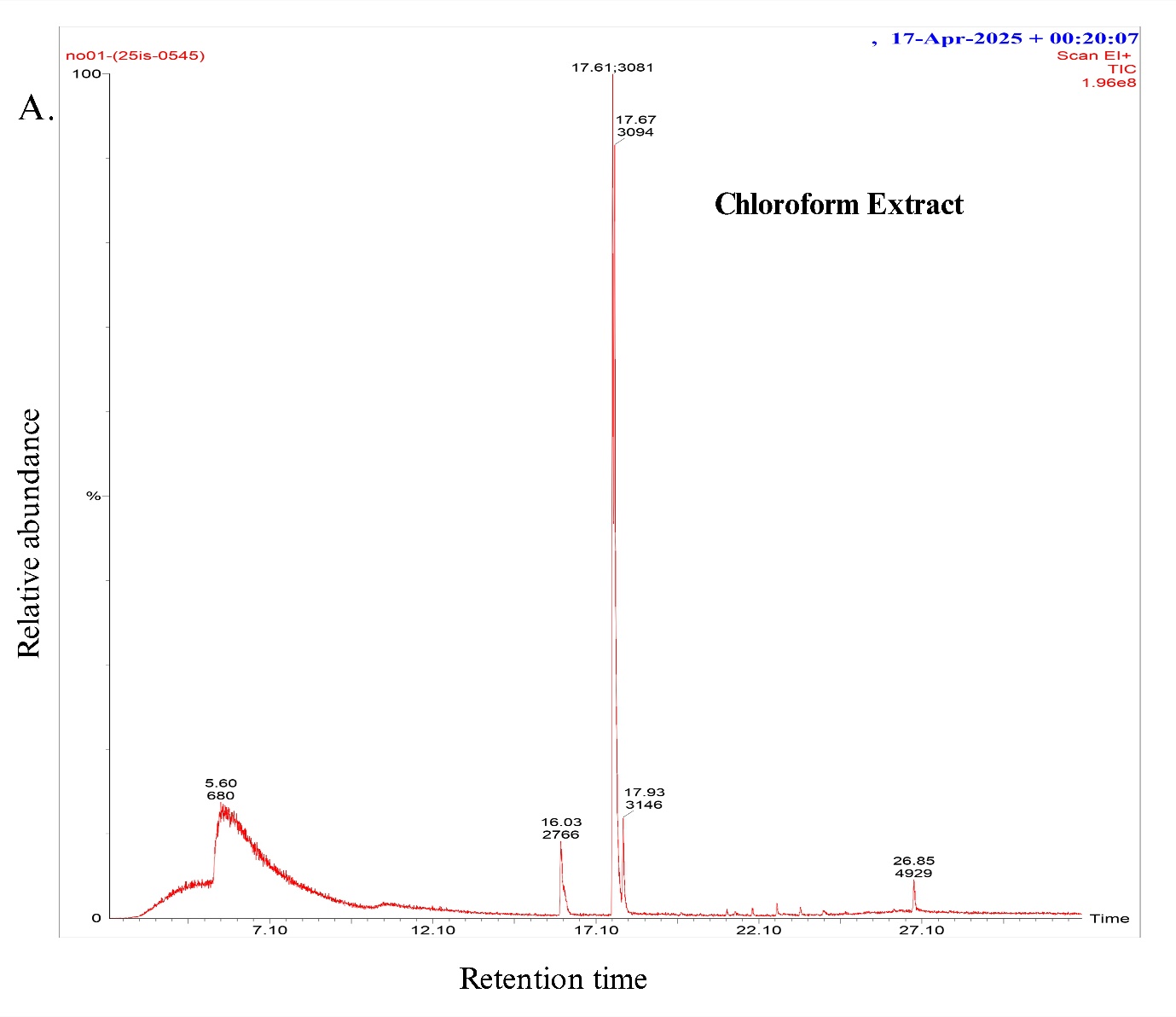

Supplement: Supplementary file 1 [file Supplementaryfile1.docx]
