# Supplementary material for: Protective role of Bergenia ciliata in artesunate-induced nephrotoxicity: a translational pharmacology study integrating in-silico and experimental evidence
Source: Front Pharmacol. 2026 Jun 24;17:1862707. doi: 10.3389/fphar.2026.1862707 (PMC13341887; doi:10.3389/fphar.2026.1862707)
Supplement: Supplementary file 2 [file Supplementaryfile3.docx]

Supplementary Table1- Biochemical assessment of oxidative stress markers

| **Parameter** | **Group1 (Normal Control)** | **Group 2 (ART 150mg/kg)** | **Group 3 (ART+BCRE200mg/kg)** | **Group 4 (ART+ BCRE 400mg/kg)** | **Group 5 (ART+ Silymarin 25mg/kg)** |
| --- | --- | --- | --- | --- | --- |
| SOD (U/mg protein) | 8.2 ± 0.4 | 3.1 ± 0.3 | 5.2 ± 0.5 | 6.8 ± 0.4 | 6.1 ± 0.5 |
| CAT (U/mg protein) | 6.0 ± 0.4 | 2.0 ± 0.2 | 4.1 ± 0.3 | 5.8 ± 0.3 | 5.0 ± 0.4 |
| GSH (U/mg protein) | 25.0 ± 1.5 | 11.5 ± 1.2 | 17.0 ± 1.4 | 22.0 ± 1.5 | 19.5 ± 1.6 |
| GPx (U/mg protein) | 7.8 ± 0.4 | 2.5 ± 0.3 | 4.5 ± 0.4 | 6.5 ± 0.4 | 5.0 ± 0.5 |
| **Lipid peroxidation** | | | | | |
| MDA (U/mg protein) | 2.8 ± 0.3 | 6.9 ± 0.4 | 5.2 ± 0.4 | 3.2 ± 0.3 | 6.0 ± 0.4 |

Supplementary Table2 -Effect of *Bergenia ciliata* root extract on plasma renal function biomarkers

| **Parameter** | **Group1 (Normal Control)** | **Group 2 (ART 150mg/kg)** | **Group 3 (ART+BCRE200mg/kg)** | **Group 4 (ART+ BCRE 400mg/kg)** | **Group 5 (ART+ Silymarin 25mg/kg)** |
| --- | --- | --- | --- | --- | --- |
| Urea (mg/dl) | 44.0 ± 3.5 | 115.0 ± 6.0 | 88.0 ± 0.5 | 62.0 ± 4.0 | 80.0 ± 5.5 |
| Uric acid (mg/dl) | 3.8 ± 0.4 | 12.0 ± 0.8 | 7.2 ± 0.6 | 6.5 ± 0.5 | 6.2 ± 0.5 |
| Creatinine (mg/dl) | 25.0 ± 1.5 | 2.15 ± 0.15 | 1.30 ± 0.12 | 0.82 ± 0.08 | 1.40 ± 0.14 |
